# Peripheral Artery Disease and Abdominal Aortic Aneurysm: The Forgotten Diseases in COVID-19 Pandemic. Results from an Observational Study on Real-World Management

**DOI:** 10.3390/medicina57070672

**Published:** 2021-06-29

**Authors:** Francesco Natale, Raffaele Capasso, Alfonso Casalino, Clotilde Crescenzi, Paolo Sangiuolo, Paolo Golino, Francesco S. Loffredo, Giovanni Cimmino

**Affiliations:** 1 Vanvitelli Cardiology and Intensive Care Unit, Monaldi Hospital, 80131 Naples, Italy; paolo.golino@unicampania.it (P.G.); francesco.loffredo@unicampania.it (F.S.L.); 2 Department of Translational Medical Sciences, Section of Cardiology, University of Campania “Luigi Vanvitelli”, 80131 Naples, Italy; raffaelecapasso@outlook.com (R.C.); giovanni.cimmino@unicampania.it (G.C.); 3 Vascular Surgery Unit, Monaldi Hospital, 80131 Naples, Italy; alfonso.casalino@ospedalideicolli.it (A.C.); clotilde.crescenzi@ospedalideicolli.it (C.C.); paolo.sangiuolo@ospedalideicolli.it (P.S.); 4 Molecular Cardiology, International Centre for Genetic Engineering and Biotechnology, 34149 Trieste, Italy; 5 Cardiology Unit, Policlinico Vanvitelli, 80138 Naples, Italy

**Keywords:** cardiovascular risk, peripheral artery disease, abdominal aortic aneurysm, lipid profile, COVID-19

## Abstract

*Background and Objectives*: It is well established that patients with peripheral artery disease (PAD) as well abdominal aortic aneurysm (AAA) have an increased cardiovascular (CV) mortality. Despite this higher risk, PAD and AAA patients are often suboptimality treated. This study assessed the CV profile of PAD and AAA patients, quantifying the survival benefits of target-based risk-factors modification even in light of the COVID-19 pandemic. *Materials and Methods*: PAD and AAA patients admitted for any reason to the Vascular Unit from January 2019 to February 2020 were retrospectively analyzed. Biochemical and CV profiles as well as ongoing medical therapies were recorded. Benefits of CV risk-factors control were estimated using the SMART-REACH model. A follow-up visit during the year 2020 was scheduled. *Results*: A total of 669 patients were included. Of these, 190 showed AAA and 479 PAD at any stage. Only 54% of PAD and 41% of AAA patients were on lipid-lowering drugs with non-optimal low-density lipoprotein (LDL) levels for most of them. A better control of all modifiable CV risk-factors based on the current guidelines would offer an absolute risk reduction of the mean 10-year CV risk by 9% in PAD and 14% in AAA. Unfortunately, the follow-up visit was lost because of COVID-19 limitations. *Conclusions*: Lipid profiles of PAD and AAA patients were far from guideline-based targets, and medical management was suboptimal. In our center, the COVID-19 pandemic impacted on the strict surveillance required in these very high-risk patients. The achievement of guideline-based therapeutic targets would definitively confer additional significant benefits in reducing the CV risk in these patients.

## 1. Introduction

Peripheral artery disease (PAD) is part of the atherosclerotic process, showing high prevalence, mortality, and morbidity in the general population [1,2]. It has been defined as a real pandemic, reflecting how systemic atherosclerosis may afflict millions of people worldwide [3]. Specifically, when compared with the known HIV/AIDS pandemic that afflicts over 34 million patients, PAD counts 202 million people with a much higher mortality rate due to cardiovascular events, mainly myocardial infarction and stroke [4,5]. It has been established that PAD patients are carriers of multiple vascular diseases (coronary artery [CAD] as well as cerebrovascular disease) [5,6,7], with a need for intensive and aggressive preventive strategies. Although PAD patients are at very high cardiovascular risk, as pointed out by the latest European Society of Cardiology (ESC) guidelines [8]), this disease is generally underdiagnosed and undertreated [9] compared with CAD patients [10]. Abdominal aortic aneurysm (AAA) is another atherosclerotic-based disease [11]. The presence of AAA is considered an equivalent risk factor as CAD [10]. It has been shown that patients affected by AAAs of any size have a risk of major cardiovascular events (MACE, including cardiovascular death or non-fatal acute myocardial infarction [MI]) that is greater than 20% at 10 years, with an additional risk of aneurysm rupture [11,12]. This high risk of MACE is mainly attributed to the presence of coronary and vascular disease, mostly asymptomatic [12,13]. Both classes of patients also require a strong surveillance of the modifiable risk factors because of their important impact on the health system and public economy. However, the recent COVID-19 pandemic [14]—because of its rapid spreading, mortality rate even for cardiovascular complications [15,16], and difficult management of non -COVID patients—has deeply changed current medical practice. In this single-center observational study, we aimed at evaluating the management of dyslipidemia and antithrombotic therapy in a population affected by PAD and AAA in 2020, at the time of SARS-CoV2 diffusion. The primary aim of the present study was to evaluate the current medical management of the modifiable major cardiovascular risk (CVR) factors in PAD as well as in AAA patients in light of the latest European Society of Cardiology (ESC) guidelines [8], with particular attention to the lipid profile achieved in these classes of patients and their antithrombotic therapy in a “real world” scenario. A secondary aim was to calculate the risk of MACE based on the clinical conditions and laboratory data (mainly lipid profile and antithrombotic drugs) of this population at the time of observation and the same risk of such events if the main therapeutic targets were achieved. Finally, the impact of COVID-19 on the management of these patients in our center was also discussed.

## 2. Materials and Methods

### 2.1. Patients Selection

This was a retrospective, cross-sectional, single-center study involving a total of 669 patients selected from those being admitted for any reason (office visit, scheduled or urgent admission) to the Vascular Surgery Unit of the Monaldi Hospital in Naples from January 2019 to February 2020. Clinical and biochemical profiles were collected. Patients with the following diagnoses were included:

- Intermittent claudication: IIA or mild claudication (free walking distance > 200 m) and IIB moderate-severe claudication (free walking distance < 200 m).
- Critical limb ischemia (stage III or IV) or the presence of pain at rest, loss of tissue, gangrene.
- Abdominal aortic or peripheral aneurysm (iliac, femoral, and/or popliteal).
- Asymptomatic or symptomatic carotid stenosis, assessed by imaging as candidates to surgical therapy (degree of stenosis ≥ 70%) if PAD or AAA was present.

Diagnosis was performed by a vascular surgeon based on a clinical examination and instrumental tools performed during outpatient visits or hospitalization according to the current guidelines [17,18].

### 2.2. Biochemical and Clinical Evaluation

Biochemical profile was obtained through the E-archive of the Clinical Biochemistry Laboratory of Monaldi Hospital. Lipid profile (total cholesterol, LDL-C, high density lipoprotein [HDL]-C, triglycerides) [8] and glucose levels were evaluated. The lack of laboratory data was not considered as an exclusion criterion. Clinical status, diagnoses, and ongoing medical therapies (especially antithrombotic therapy) of the enrolled patients were collected from the E-records and discharge summary.

### 2.3. Cardiovascular Risk Estimation

For risk quantification and evaluation of the therapeutic benefits in terms of cardiovascular events at 10-years, the SMART Risk Score model (second manifestations of arterial disease) was applied [19]. It is a validated statistical model for estimating the risk of cardiovascular events in patients already at high risk, such as CAD, stroke, PAD, AAA, as well as polyvascular disease, even previous carotid surgery [19]. This model has already been used in a similar cohort of patients [9]. The SMART-REACH model is available online (https://www.u-prevent.com (accessed on 30 November 2020)).

The SMART risk score was chosen because estimating overall CVR using other risk assessment systems (e.g., SCORE, Framingham) is not recommended in patients already at high or very high CVR, such as history of vascular events, diabetes, familial dyslipidemia, chronic kidney failure, etc. [20].

### 2.4. Follow Up and COVID-19 Restrictions

A regular follow-up was scheduled for all the selected patients (10 patients per day, twice a week). Unfortunately, because of Sars-CoV2 infection and high hospital pressure, a first lockdown was announced in Italy on 9 March 2020 and for the following 69 days all non-essential activities were stopped, including follow-up visits. Despite the pandemic, in June 2020 some hospital services were reopened, and new ways of working were developed to continue to reduce the risk of COVID-19. Specifically, five patients per week were scheduled for an office visit with a 1 h delay between each admission due to the in-hospital COVID-19 restriction. This activity started at the end of June and continued for the subsequent 11 weeks, accounted 55 scheduled patients.

### 2.5. Statistical Analysis

Due to the observational nature of the study, a formal calculation of the study sample size is not applicable, and only descriptive analyses were performed. Distribution of continuous data was tested with the Kolmogorov–Smirnov and the Shapiro–Wilk test. Normally distributed variables are expressed as mean ± standard deviation (SD), whereas non-normally distributed variables are expressed as median and interquartile range (IQR). Categorical variables are reported as numbers and percentages. Normally distributed continuous data were compared using Student’s *t* test. Comparisons between categorical data were performed using the χ^2^ test. For all tests, a *p* value < 0.05 was considered statistically significant. Analyses were performed by using R version 3.5.1 (R Foundation for Statistical Computing, Vienna, Austria).

## 3. Results

### 3.1. Study Population

A total of 669 patients (mean age 72 ± 9; 21% women) were included. Clinical characteristics, medical history, and pharmacological management are summarized in Table 1.

Of these, 479 were classified as PADs. In detail, 247 patients presented with carotid stenosis (37%), symptomatic or asymptomatic, 115 patients with lower extremity arterial disease (stage IIa, IIb) (17.2%), 81 patients with critical limb ischemia (stage III, IV) (12%), 22 patients with acute lower limb ischemia (3.3%), and 14 patients with popliteal aneurysm (2.1%). A total of 190 patients reported sub-renal AAA (28.4%). As for the main cardiovascular risk factors, 87% of patients in the PAD group had systemic arterial hypertension, 41% were current or former smokers, 42% with diabetes, while 26% had a history of CAD (with previous coronary revascularization by stent implantation or bypass grafting). In the AAA subgroup, hypertension was confirmed as the main risk factor in 89% of the enrolled patients, followed by dyslipidemia (55%), smoking status (46%), or previous history of CAD (27%).

### 3.2. Medical Therapy

#### 3.2.1. Antihypertensive Treatment

Hypertensive drugs were equally distributed in both groups. In the PAD group, 29% were treated with ACE inhibitors, 27% with a beta-blocker, and 26% with a calcium channel blocker, while 35% were treated with other antihypertensive drugs (e.g., AT-antagonists, clonidine, etc.). In the AAA group, 36% of patients were taking ACE inhibitors, 31% were treated with beta-blockers, and 29% received a calcium channel blocker, while 29% were taking a treatment different from those mentioned (Table 1).

#### 3.2.2. Treatment of Dyslipidemia

Overall, 339 patients (51%) were treated with statins, of whom 238 (70%) were on atorvastatin, 14% on simvastatin, 11.5% on rosuvastatin, and 4.1% on other statins (e.g., pravastatin, lovastatin, fluvastatin). It is alarming that 48% of the study population was not on any lipid-lowering medication. Approximately 13.6% of patients receiving statins (91) were on high-intensity statin therapy (atorvastatin 40–80 mg, rosuvastatin 20–40 mg) as recommended by ESC guidelines for high-risk cardiovascular patients, such as individuals with PAD (as reported in Table 1). In patients with AAA, statins were prescribed only in 41% of cases.

**Figure 1:**
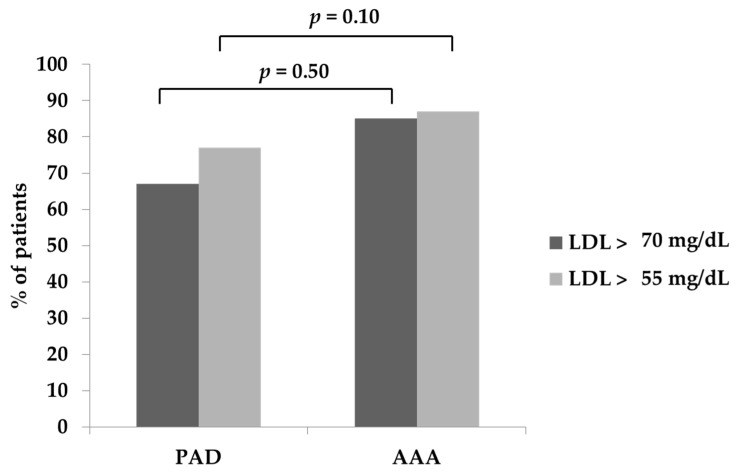
Low-density lipoproteins profile in PAD vs. AAA patients.

The mean level of total cholesterol was 159 ± 43 mg/dL, non-HDL cholesterol was 111 ± 41 mg/dL, while the mean LDL was 95 ± 33 mg/dL. Looking at the patient distribution according to lipid profile, it is interesting to note that 27.6% had non-HDL cholesterol levels greater than 130 mg/dL, with 51.1% above 100 mg/dL and 66.6% above 85 mg/dL. In terms of LDL-C target, 37% had levels ≥100 mg/dL, and 70% were above 70 mg/dL. In particular, 86% of patients had LDL-C >55 mg/dL, which is the target that should be achieved in individuals at very high CVR, such as PAD patients. Of note, 85% of PAD patients and 87% of AAA patients showed LDL-C >55mg/dL (Figure 1). The lipid profile of study population is reported in Table 2

Moreover, considering patients receiving high-dose statins, only 19 achieved the suggested target with more than 79% of patients with LDL cholesterol levels > 55 mg/dL. Similarly, among patients treated with statins at standard doses, more than 85% were far from the suggested target (Table 1).

No laboratory data were available for only 36 patients (0.5%) for either total cholesterol or LDL-C.

A complete lipid profile of the study population is reported in Table 2. Between the two groups, AAA patients showed the worst profile.

Specifically, in PAD patients, the mean total cholesterol was 156 mg/dL, non-HDL was 108 mg/dL, and LDL was 94 mg/dL, while the mean HDL was 48 mg/dL; in this subgroup, the target of 55 mg/dL of LDL was not achieved in 85% of cases, while 67% had LDL values > 70 mg/dL (Table 2, Figure 1). In AAA patients, the mean total cholesterol was 164 mg/dL, the mean non-HDL was 119mg/dL, and the mean LDL was 104 mg/dL, with all of these values significantly higher compared with PAD patients. HDL-C levels were 46 mg/dL, significantly lower compared with PAD patients. In this group, 87% of patients had LDL-C > 55 mg/dL, while in 77% LDL-C was above 70 mg/dL (Table 2, Figure 1).

Triglyceride levels did not significantly differ between the two subgroups.

#### 3.2.3. Anti-Platelet Therapy

Results from the distribution of antithrombotic drugs in the study population clearly indicate greater attention to this issue. As reported in Table 1, 1% of patients were not taking any antiplatelet or anticoagulant. Specifically, of the total PAD patients, only 52 (10.8%) were treated with clopidogrel, while 124 (25.8%) were taking daily aspirin. Dual antiplatelet therapy (DAPT: clopidogrel plus aspirin) was prescribed in 204 PAD patients (42.5%). A total of 55 patients were prescribed oral anticoagulants (11.5%) for previously diagnosed atrial fibrillation. In the AAA group, aspirin was used in 57.9% of patients, while clopidogrel in 6.3% and only in 4% of cases DAPT was prescribed. In 13.7% of patients, anticoagulants were used for preexisting diseases.

#### 3.2.4. Antidiabetic Therapy and Glycemic Targets

Of the total number of diabetic patients, the majority (61%) were treated with metformin; 31% were on insulin therapy, and 18% were taking sulfonylureas. About 20% of patients were taking other hypoglycemic agents (gliptins, repaglinide, acarbose). The glycemic targets unfortunately cannot be evaluated effectively in this study, as it was not possible to establish the modalities of individual blood collection (fasting or random). Taking into account this limitation, it might be noted that mean glucose levels of the entire study population were about 110 mg/dL, with the diabetic subpopulation averaging 136 mg/dL.

### 3.3. Impact of Risk Factor Control on Cardiovascular Risk

Since almost all the enrolled patients (661) were aged between 40 and 90 years at the time of the study, the SMART risk score was applicable.

For this purpose, the population was divided into two macro groups: PAD patients (chronic lower limb arterial disease, carotid arterial disease, etc.) and AAA patients. In the first group, the mean age was 71 ± 9.4 years with 74% males. The mean total cholesterol level was 156 mg/dL, with HDL-C 48 mg/dL and LDL-C 94 mg/dL. In light of these data and taking into account the clinical impact of PAD or AAA only, in PAD patients, the 10-year risk of cardiovascular events (MI, stroke, or CV death) was estimated to be 26%. In the second group, the mean age was 74 ± 9.4 years with 91% males. The total cholesterol averaged 164 mg/dL, with HDL-C 46 mg/dL and LDL-C mean levels 104 mg/dL. Based on these data, the 10-year risk of cardiovascular events in this subgroup, according to the SMART risk score, was estimated to be 39%.

In PAD patients the mean cardiovascular events risk reduction at 10 years, assuming the achievement of the targets suggested by the current ESC guidelines for this class of patients (LDL-C < 55 mg/dL) was 5.3% (10-year, number needed to treat [NNT] = 19), bringing the risk from the current 26% to 20.7%. A further reduction to 19% would be achieved in the presence of antithrombotic therapy (10-year, NNT 11). In AAA patients, where the estimated risk was much higher than the PAD patients, an adequate management of risk factors could lead a further benefit in terms of mean 10-year cardiovascular events as high as 9.2% (10-years NNT 11) that could reach 14% (10-year, NNT 7) if antithrombotic drugs are used.

**Figure 2:**
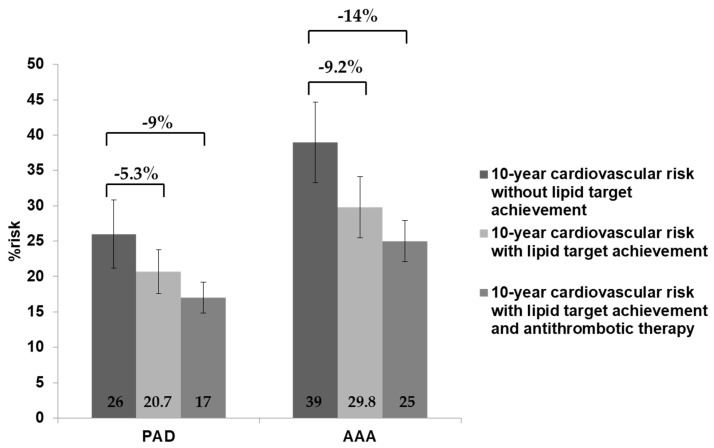
Mean 10-year cardiovascular risk estimation considering PAD or AAA alone: comparison of risk between the study population according to lipid profile and antithrombotic therapy.

These data are schematically presented in Figure 2.

Moreover, if AAA is present in the PAD population included in our analysis, the mean 10-year cardiovascular events will increase to 39%, with an expected reduction of 12.6% (10-year, NNT 8) if the lipid target is achieved and antithrombotic drugs are prescribed. Conversely, taking into account the risk profile of the AAA patients enrolled, if PAD is present, the mean 10-year cardiovascular events will be 47% with benefits accounting for a 16.6% reduction (10-year NNT 6) if guideline-based targets were achieved.

Importantly, the presence of additional risk factors (such as coronary artery diseases, cerebrovascular diseases, diabetes, and smoking status) highly increased the mean 10-year risk with a greater benefit if the ESC targets were achieved and antithrombotic therapy was optimal, as shown in Table 3.

### 3.4. Follow-Up during COVID-19 Pandemic

In June, some hospital activities reopened but with several limitations. Specifically, five patients per week were scheduled for office visit with a 1 h delay between each admission due to the in-hospital COVID-19 restrictions. However, in September, because of an increased number of Sars-CoV2 infections, a second lockdown was announced. From the end of June and for the subsequent 11 weeks, office visits were rescheduled according to the new restrictions for 55 patients that were contacted to confirm a date and time of visit. However, only 24 attended the visit because of the personal belief of COVID-19 in-hospital risk of infection and the perception that management of modifiable risk factors was not essential at the time.

## 4. Discussion

This single-center study provides a snapshot of the current management of patients with multi-district arterial disease and AAA in our institute, indicating that (1) these classes of patients are clearly undertreated, especially for the lipid profile; (2) achievement of target-based guidelines might confer additional benefits; and (3) the COVID-19 pandemic has strongly changed our medical practice because of several limitations announced to reduce the burden of pandemic, thus impairing the strict surveillance required for these patients.

By controlling the various risk factors and improving the secondary prevention therapies, as indicated by the SMART-REACH model, we could reduce MACE and disability among these very high-risk patients, thus impacting on social medicine and the public economy.

The SMART risk score can be used to estimate the 10-year risk of MI, stroke, or vascular death in individuals with clinically manifested atherosclerotic disease [21]. It is based on easy-to-measure patient clinical characteristics such as age, sex, smoking, diabetes, blood pressure, total cholesterolemia, HDL-C, LDL-C, creatinine levels, and number of cardiovascular disease sites (CAD, aortic aneurysm, PAD, etc.) [19,21]. Completion of all fields is required to estimate risk at 10-years. However, for some parameters (such as HDL-C, creatinine values, etc.) it is possible to use the average values of the population. The SMART risk score was developed in a population of patients with vasculopathy in the Netherlands who were included in the SMART (Secondary Manifestations of Arterial Disease) study [21], and the instrument was then validated externally on multiple cohorts of patients affected by vascular diseases in different countries [19,22]. Therefore, it is a statistical model applicable at the multinational level using standardized and low-cost parameters. The identification of patients at high CVR by this score should allow an appropriate therapy to be initiated as soon as possible, with favorable effects for both healthcare personnel and patients in order to re-evaluate medical therapy to achieve the suggested therapeutic targets [19,22].

The demographic distribution confirms the high incidence of these diseases in male and elderly patients (80% of patients >65 years). In our cohort, hypertension was the most common risk factor, followed by dyslipidemia, smoking status, and diabetes. In line with previous reports, our study confirms that this category of patients is inadequately treated [8], especially regarding the lipid profile and the need for constant surveillance. It is particularly alarming that about 86% of the enrolled patients did not achieve the LDL-C of 55 mg/dL suggested by the current guidelines [8], and even considering the target of 70 mg/dL [23], more than 70% of the study population was not at target. These data clearly indicate a lack of attention to the prevention of the main CVR factors in this population, especially in lipid control and antithrombotic treatment, probably due to a reduced perception of risk among the medical community at both the specialized level (vascular surgeons and cardiologists) as well as among general practitioners. It is important to emphasize that management of dyslipidemia needs a personalized approach that considers the CVR of each patient, as recommended by the guidelines [8]. It is worrying that 48% of patients were not on a lipid lowering strategy and that those on statins treatment were far from the target LDL-C. Of the total of 339 patients (51%) on statins treatment, only 91 were taking high-intensity statins, while the remaining 248 were at standard doses. Moreover, of these 91 patients receiving high-dose statins only 19 achieved the target with >79% of patients with LDL-C > 55mg/dL. Similarly, among patients being treated with standard-dose statins, >85% were far from the suggested target. The benefits of low LDL-C levels is indeed now widely accepted and supported by several studies [24,25]. In the IMPROVE-IT clinical trial, a 24% further lowering of LDL-C level when ezetimibe was combined with simvastatin was associated with additional benefits than when simvastatin was administered alone [26]. Over the course of the IMPROVE-IT study, the median time-weighted average LDL-C level was 69.5 mg/dL in the simvastatin-monotherapy group and 53.7 mg/dL in the simvastatin–ezetimibe group. This LDL-C level was associated with a 2% absolute risk reduction and a significant reduction of 6.4% of the primary composite endpoint of cardiovascular death, non-fatal MI, unstable angina requiring hospitalization, coronary revascularization (30 days after randomization), and stroke [26].

The importance of a specific antiplatelet therapy is suggested by the CAPRIE study (Clopidogrel versus Aspirin in Patients at Risk of Ischaemic Events) [27], where clopidogrel was superior to aspirin in a subgroup of PAD patients, specifically in those with distal arterial disease of the lower limbs, showing a significant reduction in mortality and morbidity. Based on the available literature, the current NICE guidelines suggest the use of clopidogrel 75 mg in PAD patients, unless contraindicated [28,29]. In our study population, clopidogrel was occasionally used (only 15% of PAD patients). Interestingly, prescription of aspirin was much higher, with up to 56% of PAD patients being treated. These data clearly indicate that even for antiplatelet therapy, PAD patients are inadequately treated.

### 4.1. Estimation of Cardiovascular Risk, Potential Benefits of Cholesterol-Lowering Therapy, and New Antithrombotic Strategy

The European Society of Cardiology suggest the use of SMART risk score [22] to assess CVR in selected population, such as PAD and AAA patients. In PAD patients, the 10-year risk of cardiovascular events was estimated to be 26%, confirming the high-risk of this group. Considering all other clinical conditions being equal, if PAD patients achieved the LDL-C target suggested by the ESC guidelines [8], the CVR at 10-years would be reduced by 5.3%. The benefit achieved by the optimization of cholesterol-lowering therapy was shown to be even higher in AAA patients, where there was a 10-year 39% risk of events, with an estimated reduction of 9.2%. These data strongly confirm that the optimization of cardiovascular secondary prevention in this class of patients is of great importance. Specifically, the achievement of cholesterol target levels affects positively the occurrence of MACE with a significant impact on survival and quality of life, as well as global health and social spending [30]. It is known that development of PAD is also associated with high disability since even claudication can compromise patient autonomy in daily activities, leading to the need for ongoing assistance [1,31,32]. In the most serious cases, the occurrence of critical ischemia or any amputation may cause permanent disabilities with further limitations of work activity and personal care [1,31,32]. The reduction of the risk in these patients is essential to limit the impact on both the health systems and the welfare system; thus, improvement of surveillance on the achievement of the current suggested targets may lead to further economic benefits despite of the costs of preventive drug therapies [33]. Recent studies also underscore the relevance of lipids in AAA progression [34] and the beneficial impact of statins on AAA patient survival [35], thus confirming the need to also better evaluate and manage the lipid profile in this class of patients.

It is important to note that while unfavorable lipid profile is associated with most cardiovascular diseases, high levels of LDL-C appear to have little impact on stroke, where this association seems indirect and not causal [36,37].

An emerging class of drugs for the management of dyslipidemias and the reduction of CVR is represented by the PCSK9 inhibitors [24]. PCSK9 functions as a negative feedback that decreases LDL-C receptor activity, which results in increased LDL-C. Thus, its inhibition leads to a decrease of total cholesterol and LDL-C by eliminating the negative feedback that induces LDL-C receptor degradation [24]. In a preselected subgroup analysis of the FOURIER study, evolocumab was shown to significantly reduce the primary endpoint (composite endpoint of cardiovascular death, MI, stroke, hospitalization for unstable angina, or coronary revascularization) in PAD patients [38]. Because of their higher risk, PAD patients had larger absolute risk reductions for the primary endpoint (3.5% with PAD, 1.6% without PAD) and the key secondary endpoint (3.5% with PAD, 1.4% without PAD). Evolocumab reduced the risk of major adverse limb events in all patients (with consistent effects in those with known PAD estimated to be around 42% [38]). Moreover, the recent data from the COMPASS trial clearly indicate that the combination of rivaroxaban 2.5 mg twice daily plus aspirin prevents adverse events, particularly stroke and cardiovascular mortality, without a significant increase in severe bleedings of clinical impact [39]. These benefits were particularly favorable in high-risk subgroups [39]. Thus, the use of these new classes of drugs may be of tremendous impact in preventing MACE and disability, thus improving patients’ outcome, social health, and the public economy.

### 4.2. The Dark Side of COVID-19 Pandemic

To improve the global management of these very high-risk patients, a regular follow up was scheduled to re-evaluate the achievement of the suggested targets for each modifiable risk factor.

The recent SARS-CoV2 infection and its spread worldwide with high pressure on hospitals have imposed several restrictions [15]. The disease has shown difficult management with several unsolved problems in terms of reconciling patient characteristics [14,15] and pharmacological strategies [40,41,42,43]. Taking in to account the in-hospital restrictions, all the appointments were rescheduled, but most of the patients lost the follow-up visit because of COVID-19 in-hospital infection risk and personal underestimation of the seriousness of the disease (PAD or AAA). Starting from September, because of a new increase in COVID-19 cases, non-essential activities were stopped again. Some strategies have been proposed to monitor selected classes of patients [44,45]. At the time of the present article’s writing, COVID-19 is still a pandemic, with ongoing moderate to severe restrictions.

### 4.3. Study Limitations

- The main limitation of this study, because of the retrospective analysis, is the missing data;
- It was not in the scope of this study to investigate the interaction between the current medical treatment, statin dose, and cholesterol level achieved. As stated above, this was a retrospective study, and thus it was not possible to know the duration of therapies for each patient.
- The research design did not allow the evaluation of patient adherence to treatment since no specific monitoring was performed.
- Lastly, regarding the study design, the present work describes only the status of our institution during COVID-19 period.

## 5. Conclusions

Our study, in line with other observations, indicates that the current management of PAD and AAA patients is suboptimal and far from the current guidelines. These classes of patients are at very high risk of MACE and need strong surveillance of their modifiable risk factors. However, the lack of attention from medical practitioners, the wrong beliefs of the patients about the need to achieve the guideline-based targets, and the COVID-19 restrictions are a dangerous mixture in the management of these “forgotten” diseases. We would like to raise awareness in the medical community that adopting guidelines and achieving targets that may be easily obtained by available drugs can improve the standard of care of PAD and AAA patients.

## Figures and Tables

**Table 1:** Clinical characteristics of the population at baseline.

Demographics	No. of Patients (*n* = 669)
Age (years)	72 ± 9
Sex ratio (M:F)	527:142
**Diagnosis at baseline**	
Carotid artery disease	247 (37%)
Intermittent claudication	115(17.2%)
Critical lower limb ischemia	81(12%)
Acute lower limb ischemia	22(3.3%)
Popliteal aneurysm	14(2.1%)
Abdominal aortic aneurysm	190(28.4%)
**Cardiovascular characteristics**	
Smoker	149(22.3%)
Ex-smoker	134(20%)
Diabetes mellitus	238(35.6%)
Systemic arterial hypertension	582(87%)
Coronary artery disease	180(27%)
**Hyperlipidemia**	
Total cholesterol ≥ 200 mg/dL	105(15.7%)
LDL cholesterol ≥ 70 mg/dL	466(70%)
LDL cholesterol ≥ 55 mg/dL	573(86%)
non-HDL > 130 mg/dL	185 (27.6%)
Triglycerides ≥ 150 mg/dL	178(26.6%)
**Cardiovascular medication**	
Aspirin	234(34.9%)
Clopidogrel	64(9.5%)
Other antiplatelet agents	9(1.34%)
Dual antiplatelet therapy	272(40.6%)
Anticoagulant	81(12%)
Angiotensin-converting enzyme inhibitor	207 (31%)
Beta-blocker	193(29%)
Calcium channel blocker	179(27%)
Other antihypertensive agents	222(33%)
**Antihyperlipidemic medication**	
Any statin	339(51%)
Atorvastatin	238(70.2%)
Simvastatin	48(14.2%)
Rosuvastatin	39(11.5%)
Other statins	14(4.1%)
High-dose statin therapy	91 (13.6%)
Ezetimibe	15 (2.2%)

**Table 2:** Lipid profile in the study population.

	PADs (*n* = 479)	AAA (*n* = 190)	*p*-Value
Total cholesterol	156 ± 42.6	164 ± 42.7	0.03
HDL cholesterol	48 ± 13.6	46 ± 13.6	0.08
LDL cholesterol	91 ± 33.4	102 ± 33.4	0.0001
Non-HDL cholesterol	108 ± 40.4	119 ± 40.4	0.002
Triglycerides	135 ± 67.6	127 ± 67.7	0.17
LDL > 55	407 (85%)	166 (87%)	0.51
LDL > 70	319 (67%)	147 (77%)	0.01

**Table 3:** CVR estimation with additional diseases and impact of ESC targets on risk reduction.

	Mean 10-Year CVR	Mean 10-Year CVR with Treatment	% Risk Reduction	10-Year NNT
PAD	26 ± 4.8	17 ± 2.2	9	11
+CAD	30 ± 4.7	20 ± 3.6	10	10
+CAD + CBD	51 ± 3.4	36 ± 2.9	15	7
+CAD + CBD + DM	59 ± 4.4	42.7 ± 3.9	16.3	6
+CAD + CDB + DM + SK	68 ± 3.8	34.7 ± 3.1	33.3	3
				
AAA	39 ± 5.7	25 ± 2.9	14	7
+CAD	43 ± 3.6	27.7 ± 3.3	15.3	7
+CAD + CBD	67 ± 4.4	47.2 ± 4.6	19.8	5
+CAD + CBD + DM	75 ± 5.4	55.1 ± 3.9	19.9	5
+CAD + CDB + DM + SK	84 ± 5.6	47 ± 4.7	37	3

PAD, peripheral artery disease; CAD, coronary artery disease; CBD, cerebrovascular disease; DM, diabetes mellitus; SK: current smoking.

## Data Availability

The data presented in this study are available on request from the corresponding author.
